# Advanced radiological work-up as an adjunct to decision in early reconstructive surgery in brachial plexus injuries

**DOI:** 10.1186/1749-7221-5-14

**Published:** 2010-07-08

**Authors:** Kasim Abul-Kasim, Clas Backman, Anders Björkman, Lars B Dahlin

**Affiliations:** 1 Department of Radiology, Skåne University Hospital, S-205 02 Malmö, Sweden; 2 Department of Hand Surgery, Skåne University Hospital, S-205 02 Malmö, Sweden; 3 Department of Clinical Sciences Malmö - Hand Surgery, Lund University, S-205 02 Malmö, Sweden

## Abstract

**Background:**

As neurophysiologic tests may not reveal the extent of brachial plexus injury at the early stage, the role of early radiological work-up has become increasingly important. The aim of the study was to evaluate the concordance between the radiological and clinical findings with the intraoperative findings in adult patients with brachial plexus injuries.

**Methods:**

Seven consecutive male patients (median age 33; range 15-61) with brachial plexus injuries, caused by motor cycle accidents in 5/7 patients, who underwent extensive radiological work-up with magnetic resonance imaging (MRI), computed tomography myelography (CT-M) or both were included in this retrospective study. A total of 34 spinal nerve roots were evaluated by neuroradiologists at two different occasions. The degree of agreement between the radiological findings of every individual nerve root and the intraoperative findings was estimated by calculation of kappa coefficient (К-value). Using the operative findings as a gold standard, the accuracy, sensitivity, specificity, positive predictive value (PPV) and negative predictive value (NPV) of the clinical findings and the radiological findings were estimated.

**Results:**

The diagnostic accuracy of radiological findings was 88% compared with 65% for the clinical findings. The concordance between the radiological findings and the intraoperative findings was substantial (К = 0.76) compared with only fair (К = 0.34) for the clinical findings. There were two false positive and two false negative radiological findings (sensitivity and PPV of 0.90; specificity and NPV of 0.87).

**Conclusions:**

The advanced optimized radiological work-up used showed high reliability and substantial agreement with the intraoperative findings in adult patients with brachial plexus injury.

## Introduction

The most common cause of closed brachial plexus injuries in adults is a motorcycle accident (70%) [1,2]. The generally agreed mechanism of a brachial plexus injury is traction stress on the plexus as the head and the shoulder are forced apart [3]. Up to 2/3 of high energy brachial plexus injuries may need surgical intervention [2]. Thus, the preoperative planning to determine type, level and extent of the injury is crucial for optimal selection of patients that benefit from surgical reconstruction and to plan the surgical procedure. Early reconstructive surgery of nerve injuries encourages rapid regeneration and repair [4-7]. Neurophysiological tests may not reveal the extent of injury at the early stage [7]. Therefore, the role of imaging studies performed early has become increasingly important.

The choice of the radiological modality in the work-up of brachial plexus injury has been continuously changed in last decades. Although myelography was the reliable [8] and the most used methods in the radiological work-up of brachial plexus injuries prior to the era of sectional imaging, its use nowadays should only be restricted to patients with contraindication to magnetic resonance imaging (MRI). Nowadays, MRI is the imaging method of choice in the work-up of brachial plexus injuries [9]. New MR sequences, e.g. 3D CISS (3-dimensional constructive interference in steady state), enable acquisition of thin slices with the possibility to perform reconstruction in three different planes and hopefully can contribute to increase the diagnostic accuracy. The disadvantages of MRI are the long acquisition time for every individual sequence and the sensitivity to movement, and thus demand for the patient to lay still. New MRI technique that recently has been recommended in the work-up of brachial plexus injury is the diffusion weighted MR neurography [10]. However, the main limitation of this technique is lack of depiction of cervical nerves above the level of the C5 nerve. Another radiological modality that is used in the evaluation of brachial plexus is the computed tomography following myelography (CT-M). As CT-M is invasive and means exposing these, often young, patients to high doses of ionising radiation, this type of imaging should also be reserved to patients with contraindication to MRI. A new modality that recently showed high feasibility in the assessment of cervical nerve roots is Bezier surface technique, which enables reformatting volumetric data obtained at CT-myelography to depict the individual nerve root in a single image [11,12]. However, most of these modalities are new and their role in the work-up of brachial plexus injury is not yet well established.

The main purpose of the radiological examination prior to brachial plexus surgery is to determine the location of the injury in relation to the dorsal root ganglion and categorize injuries into preganglionic avulsion or postganglionic rupture or stretching. The aim of this study was to evaluate the accuracy of the radiological findings and the clinical signs with the intraoperative findings in adult patients with brachial plexus injuries.

## Methods

Seven consecutive male patients with brachial plexus injuries who underwent MRI, CT-M or both were included in this retrospective analysis. The median age for the patients was 33 years (mean age 29 ± 17 years; range 15-61 years). All patients were evaluated by the same surgeons preoperatively and the extent of the lesion was determined clinically (e.g. evaluation of pain, Tinel sign, presence of Horner syndrome, loss of muscle function and sensory deficit). Preoperatively, all patients underwent MRI, within 15 days of injury in average (median 7 days). Two patients had also been examined using CT-M because of motion artefacts in MRI in one patient (patient No 6) and because of hematoma and fibrosis at the root exit which resulted in a significant signal drop on MRI in another patient (patient No 2). One patient (patient No 5) underwent MRI two times (one at the hospital where the patient was initially admitted and one in our institution). All patients were examined with sagittal T1-weighted images (WI), axial T1 WI, axial T2 WI, axial turbo flash (TF) gradient echo images, and coronal short TI inversion recovery (STIR)-images. In three cases (case 1, 2 and 4) the patients were also examined with a dual excitation sequence called 3D CISS. The images were evaluated at two different occasions, one at the time of injury and one at the time of analysis of this study. In cases of disagreement the final results were reached by consensus at joint evaluation of two radiologists. The reader was blinded to the clinical and the intraoperative findings. The radiological signs of brachial plexus injuries sought for were the following: (a) signal changes in the spinal cord near the nerve root exit, (b) bleeding near the nerve root exit, (c) failure of visualisation of the nerve root (dorsal, ventral or both), (d) discontinuity in the course of the nerve root (dorsal, ventral or both), (e) CSF leakage along the nerve root, and (f) pseudomeningocele. In 6 patients the spinal roots C5-T1 were examined and in the seventh patient only C6-T1 were examined. In all patients, the brachial plexus lateral to ganglion (trunks, divisions, and cords) was also evaluated. For the purpose of evaluation the aforementioned structures (trunks, divisions, and cords) were considered as postganglionic plexus. The total number of the evaluated spinal nerve roots was 34. The agreement between the radiological findings of every individual spinal nerve root and the preoperative findings of each root at the time of the surgical exploration was estimated.

All patients were operated on by the same surgeons in average 26 days (median 17 days) after the injury when the extent and location of the lesion was determined. All patients were operated on in general anaesthesia with a supraclavicular approach extending along the infraclavicular plexus, usually using an osteotomy of the clavicle, through a longitudinal incision in the deltopectoral groove from approximately the middle of the clavicle to the cranial border of the tendon of pectoralis major. Appropriate nerve reconstructive procedures were done based on the findings in the individual patients.

The study was approved by the local Ethics committee of Lund University. The study was done in accordance with the Helsinki declaration.

### Statistical analysis

Statistical analysis was performed using SPSS 17. The degree of agreement between the clinical findings and radiological findings of every individual spinal nerve root on one hand and the intraoperative findings on the other hand was estimated by cross tabulation and calculation of kappa coefficient (K-value). The interpretation of kappa values was done according to the method proposed by Landis [13]. A 2-way contingency table was generated comparing the clinical findings and radiological findings on one hand with the operative findings on the other hand. The contingency table was used to calculate the accuracy, sensitivity, specificity, positive predictive value (PPV) and negative predictive value (NPV) of the clinical findings and the radiological findings with the operative findings as a gold standard.

## Results

### Patients' characteristics

Motor cycle accident was the cause of the injury in five patients. The remaining patients were subjected a ski accident (n = 1), and a trauma of a falling tree (n = 1) (Table 1). Five patients showed injuries of the right sided brachial plexus. Four out of seven cases were clinically suspected to have total damage of plexus brachialis (C5-T1-injury). Six patients had other serious associated injuries of which three were suspected to have total damage of the brachial plexus (Table 2). The preoperative clinical signs of the patients are summarized in Table 1. The clinical signs of the extent of the lesion (pre- or postganglionic injury) showed a suspicion of preganglionic (based on no Tinel sign, character of the pain, presence of Horner syndrome) or of a postganglionic injury (presence of Tinel sign, remaining motor function in serratus anterior muscle).

**Table 1:** Patient characteristics and summary of the clinical, radiological and intraoperative findings in seven patients with a traumatic brachial plexus injury.

No	Age (yr)	Injury mechanism	Imaging modality	Clinical findings	Imaging findings	Operative findings	Side affected
1	15	MC	MRI	C5-C6	C6	C5-C6	Right
2	15	MC	MRI + CT-M	C5-T1	C5-C7	C5-C7	Right
3	34	MC	MRI	C5-T1	Postgangl. rupture at the level of the cord	Postgangl. rupture at the level of the cord	Right
4	34	MC	MRI	C5-T1	C6-T1,	C5-T1	Left
					C5 not included on axial images		
5	14	Ski injury	MRI	C5-C6	C5-C6	Intact roots (axonotmesis)	Right
6	61	Falling tree	MRI + CT-M	C5-C8	C6	Postgangl. C5, avulsion C6	Left
7	33	MC	MRI	C5-T1	C5-C8	C5-C8	Right

No = Patient number. yr = year. MRI = Magnetic Resonance Imaging. CT-M = Computed Tomography- Myelography. MC = Motor cycle. C indicates cervical roots and T thoracic roots. Postgangl. = Postganglionic injury.

**Table 2:** Time between injury and radiological examination and surgery in seven patients with a traumatic brachial plexus injury expressed in days.

No	Injury-Radiological work-up	Injury-Surgery	Associated injury
1	5	16	Metacarpal V fracture
2	7	17	None
3	26	48	Metatarsal injury, ankle fracture, radius and ulna fractures, supracondylar humerus fracture, and radial nerve injury at elbow level.
4	23	27	Shoulder dislocation, metacarpal II-V fracture, radius fractures, and ligament injury left knee.
5	4	14	Lung contusion, skull base fracture, mandibular fracture, orbital fracture.
6	33	42	Hemo-/pneumothorax, scapular-, clavicular-, and rib fractures
7	4	17	Clavicle fracture, unstable T12 fracture, multiple rib fractures with flail chest, hemo-pneumothorax, compartment syndrome forearm, metacarpal V fracture, and right subclavian artery injury.

### Radiological work-up

**Figure 1:**
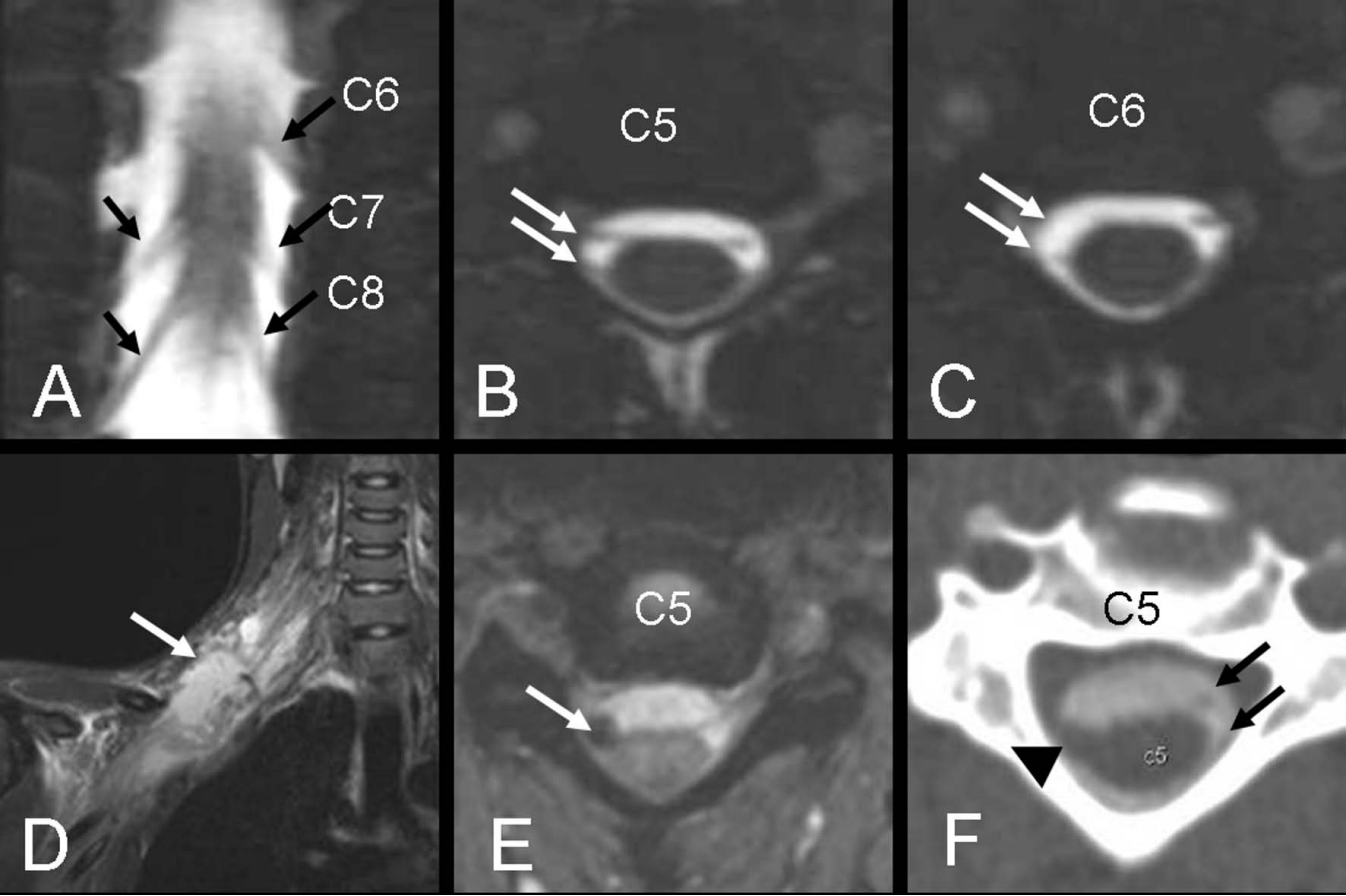
**(A-C) MRI 3D CISS of patient No 1**. The Coronal image (A) shows avulsion of C6 root on the right side. The intact roots are marked with arrows. Axial images (B-C) show normal C5 roots (arrows, B) and avulsion of C6 roots on the right side (arrow, C). However, exploration revealed avulsion of both C5 and C6 on the right side (false negative MRI-finding at C5). (D-F) Images of patient No 2. The coronal STIR (D) shows edema around the supra- and infraclavicular plexus. (E) Axial turbo flash image shows extremely low signal at the C5 root exit indicating bleeding. (F) Axial CT-M shows hematoma at the site of dorsal root exit (arrow head) and absence of ventral root. Black arrows show the normal C5 roots on the left side. Similar findings were revealed at the level of C6 and C7. MRI findings were concordant with the intraoperative findings.

Out of 34 spinal roots subjected for radiological evaluation, the diagnosis was the same as the intraoperative diagnosis for 30 of the explored nerve roots. This resulted in diagnostic accuracy of 88%. The concordance between the radiological findings and the intraoperative findings was substantial (К value 0.76; 95% CI 0.54-0.98). There were two false positive and two false negative radiological findings, which resulted in sensitivity and positive predictive value of 0.90 (95% CI 0.76-0.96), and specificity and negative predictive value of 0.87 (95% CI 0.70-0.95), (Table 3). The accuracy of clinical diagnosis was 65% (in 22 of the 34 explored nerve roots the clinical diagnosis was the same as the intraoperative findings), which resulted in only fair agreement (К value 0.34; 95% CI 0.11-0.56). At the surgical exploration, 12 roots that the clinical examination raised a suspicion of root injury were found to be intact (false positive clinical findings). This resulted in a specificity of 0.56 and positive predictive value of 0.37 (Table 3). Figure 1 show examples of the radiological findings in two different patients included in this study.

**Table 3:** 2-way contingency table comparing the radiological and clinical findings on one hand with intraoperative findings on the other hand.

				P-value	Sensitivity	Specificity	PPV	NPV
**Root injuries on MRI**								

		No	Yes					
Root injuries	No	17	2					
at operation	Yes	2	13	<0.001	0.90 (0.76-0.96)	0.87 (0.70-0.95)	0.90 (0.76-0.96)	0.87 (0.70-0.95)

**Root injuries suspected clinically**								

Root injuries	No	7	12					
at operation	Yes	0	15	0.011	1 (0.69-1)	0.56 (0.48-0.56)	0.37 (0.25-0.37)	1 (0.86-1)

PPV indicates positive predictive value. NPV indicates negative predictive value

### Operative findings

In two cases (cases 4 and 7) there was a need for division of the clavicle in order to visualize all nerve endings and roots. The roots were evaluated as avulsed or ruptured. The texture and looseness of the nerve roots were considered in the decision as to if the nerve could possibly be avulsed but still in the spinal canal or intact. In cases of scarred tissue over the plexus the area was explored and meticulously dissected and ruptures were defined.

The clinical signs at evaluation of the patients indicated upper trunk injuries in case 1, which was confirmed at surgery as we found C5 and C6 avulsions. On exploration, case 2 showed C5-C7 rupture and the lower roots were soft in texture, but were considered partially injured. However, at a later follow-up there was M3 for flexor digitorum profundus (FDP) muscles and M2 for flexor pollicis longus (FPL). In case 3, the preoperative findings showed partial function in C5 and C7, while the other roots were considered ruptured or avulsed. At surgery there was rupture of the whole plexus at the infraclavicular level. In case 4, a total avulsion of the entire plexus was found. Case 5 showed clinical signs of partial rupture in upper trunk, however, at surgery the plexus was found intact (axonotmesis). In case 6, there were, apart from clinical signs of upper trunk rupture, rupture or avulsion of C7-C8, while a partial function was seen in T1 innervated muscles. At surgery C5 was found ruptured and C6 avulsed, while C7 and C8 were evaluated as intact. At a later follow up there was some recovery in the forearm flexors and M1-2 in wrist extensors and extensor pollicis longus (EPL), indicating a partial rupture in the latter nerve roots. In case 7, there was rupture of C5, C6 and C8, and avulsion of C7 while T1 was not visualized.

## Discussion

**Figure 2:**
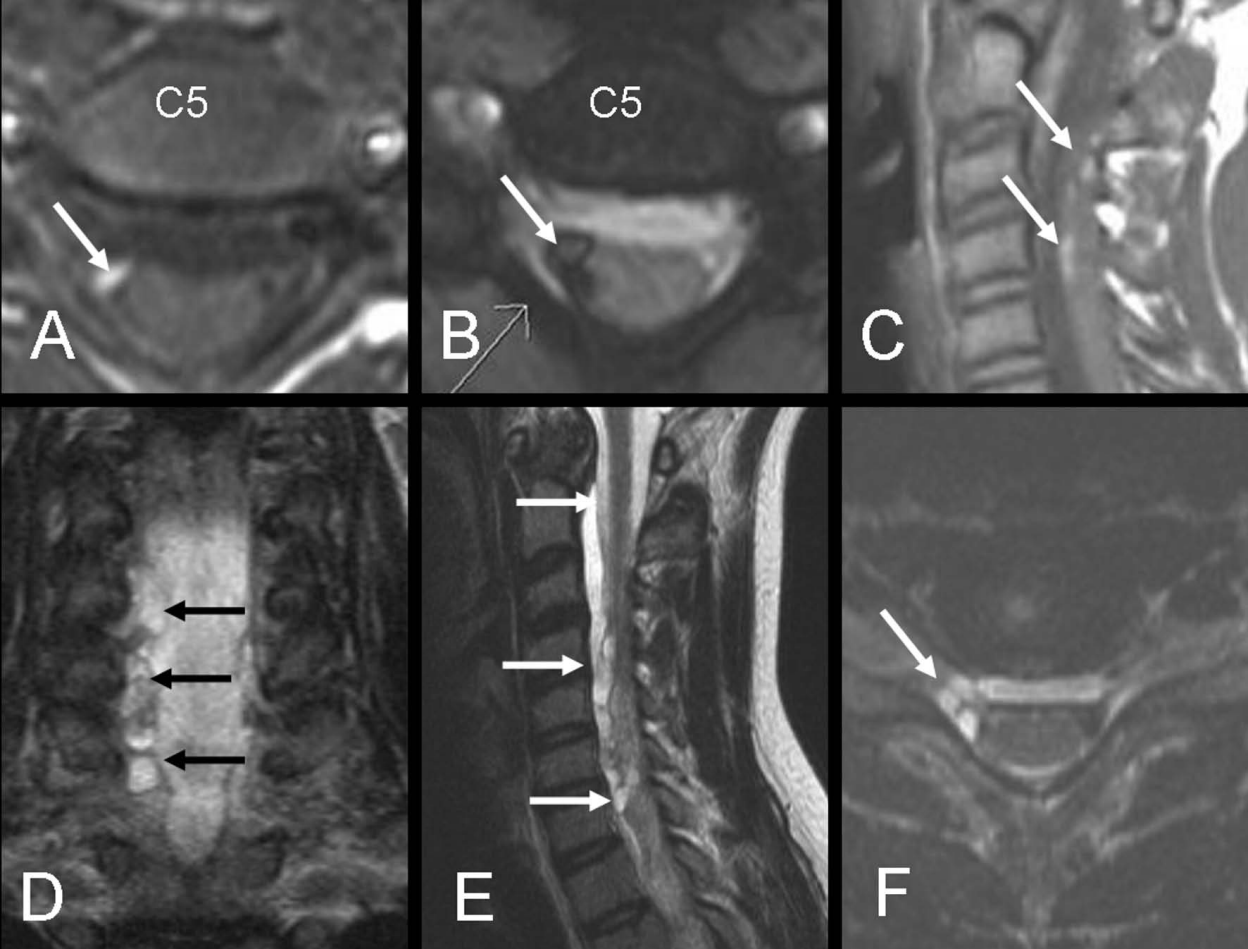
**(A-B) axial T1WI and turbo flash image. **(C) sagittal T1WI of the initial MRI of patient No 5 show methemoglobin at the C5 root exit with high signal intensity on T1WI and extremely low signal intensity on turbo flash images (arrows). Prior to surgery a new MRI (D-F) coronal STIR, sagittal T2WI and axial turbo flash image showed development of pseudomeningocele (intradural cysts) along the nerve roots at several levels (arrows). Despite these findings the roots were found to be intact (axonotmesis) on exploration (false positive MRI-finding).

The present study showed that the radiological work-up in adult patients with brachial plexus injuries contributed to a better preoperative diagnosis with increased diagnostic accuracy as compared to a clinical examination alone and routine MRI, which may be useful for the surgeon for the preoperative decision making of possible reconstruction possibilities. The radiological diagnostic accuracy was clearly better than the clinical diagnostic accuracy. This may depend on the fact that patients with brachial plexus injuries usually are severely injured with multiple associated injuries that make the clinical evaluation difficult to perform and interpret. Furthermore, the patients may be severely injured or treated in a respirator making a proper clinical evaluation impossible to perform. Radiological work-up showed a high accuracy (88%), a high sensitivity (90%), and a high specificity (87%) compared to the intraoperative findings. Carvalho et al reported a diagnostic accuracy of the preoperative CT myelography and MRI of 85% and 52%, respectively [14], while Hems et al reported a sensitivity of 81% for MRI [15]. We believe that higher accuracy and sensitivity in our study, compared with the aforementioned studies, depends on the followings: (a) use of new MR-sequences, such as 3D CISS, which enables acquisition of thin slices, reconstruction in three planes and generation of images that resemble myelography, (b) use of gradient echo sequences (turbo flash), which is very sensitive to minimal bleedings at e.g. the nerve root exit, and (c) inclusion of CT-myelography whenever MRI provides insufficient preoperative data. Specificity and PPV could have been increased to 1 if there were no false positive result (patient No 5). However, such clear and distinct MRI-findings that were radiologically confirmed in case 5 (hematoma at the root exit C5 and C6 and subsequent development of pseudomeningocele) should be reported and regarded as signs highly suggestive of root avulsion (Figure 2).

Of course, no study is without limitations. Two major limitations of this study are its retrospective nature and the limited number of patients included in the analysis. The limited number of patients may have make it difficult to keep the radiological reader totally blinded as there is a small, but existing, possibility that the reader remembered the findings in some of the evaluated images. However, the evaluation of the images in our study was focused on the individual roots rather than on individual patients. We believe that our findings of high reliability of the optimized radiological work-up with addition of special MRI sequences or performance of CT-myelography to reveal the precise extent of the brachial plexus injury is worth to report. In addition, the analyses have been done by the same surgeons and radiologist in all patients which is strength of the study.

We performed all brachial plexus explorations and reconstructions early in most of the cases (within 27 days in 5 out of 7 cases); a decision based on neurobiological knowledge indicating that alterations after injury in neurons and non-neuronal cells are rapid with respect to cell death and signal transduction. Motor and sensory neurons die after a nerve injury [4,5]. In addition, a nerve injury induces rapid, sometimes transient, upregulation of transcription factors in various signal transduction pathways, a phenomenon which can not be utilized if nerve repair or reconstruction is delayed and may lead to impaired axonal outgrowth [16-20]. The neurobiological data is supported by a recent clinical study indicating better functional outcome if brachial plexus injuries in adults are reconstructed without a long delay [1,6].

As our radiological work-up showed high accuracy, sensitivity, and a high specificity as well high concordance with the intraoperative findings, we strongly recommend the use of new MR-sequences, such as 3D CISS (3-dimensional constructive interference in steady state) or complementary CT-M, to reveal the extent of the brachial plexus injury.

## Conclusion

We conclude that radiological investigation plays an important role in the preoperative work-up of adult patients with a brachial plexus injury, where early reconstruction of the injury may be decisive for an improved outcome. Advanced and optimized radiological work-up of this study showed high reliability and substantial agreement with the intraoperative findings. We strongly recommend the use of new MR-sequences, such as 3D CISS (3-dimensional constructive interference in steady state) or addition of CT-myelography, to precisely reveal the extent of the brachial plexus injury.

## Competing interests

The authors declare that they have no competing interests.

## Authors' contributions

KAK performed the radiological evaluation. All surgery has been done by CB, AB, and LBD. All authors have equally in different ways contributed to the manuscript. All authors read and approved the final manuscript.
